# Editorial: Computational intelligence in personalized medicine

**DOI:** 10.3389/fphar.2022.1046271

**Published:** 2022-10-12

**Authors:** Yuanpeng Zhang, Khairunnisa Hasikin, Samiappan Dhanalakshmi, Yan Chai Hum, Khin Wee Lai

**Affiliations:** ^1^ Department of Medical Informatics, Nantong University, Nantong, China; ^2^ Department of Biomedical Engineering, Faculty of Engineering, Universiti Malaya, Kuala Lumpur, Malaysia; ^3^ Department of Electronics and Communication Engineering, Faculty of Engineering and Technology, SRM Institute of Science and Technology, Kattankulathur, India; ^4^ Department of Mechatronics and Biomedical Engineering, Lee Kong Chian Faculty of Engineering and Science, Universiti Tunku Abdul Rahman, Kajang, Malaysia; ^5^ Department of Biomedical Engineering, Faculty of Engineering, Universiti Malaya, Kuala Lumpur, Malaysia

**Keywords:** computational intelligence, personalized medicine, machine learning, deep learning, clinical decision support

Emerging developments and innovations in digital healthcare technologies, also known as digital transformation, may drastically enhance current healthcare operations. Among advanced technologies, artificial intelligence, particularly computational intelligence, has proven successful as a technology trend contributing to the digital transformation of the healthcare and medical industries. For example, Zhang et al. proposed a multimodal neuroimaging embedding feature selection and fusion method for the multiclass diagnosis of Alzheimer’s disease (Zhang et al., 2021). Zhang et al. also proposed an imbalance classification framework and a high-generalizable classifier for distant metastases prediction of advanced nasopharyngeal carcinoma (Zhang et al., 2022). In this research topic, the publications have been rigorously peer-reviewed by external reviewers with strong backgrounds in computational intelligence research. These high-quality publications provide efficient and precise intelligent algorithms for the field of personalized medicine.

Among these publications, two research groups focused on personalized medicine in cancer, and each has described a very good computational intelligent algorithm. Wang et al. (Xu et al.) proposed a semi-supervised learning framework based on Vision Transformer (ViT) for the diagnosis of breast cancer, which unifies both supervised and consistency training to enhance the robustness of the model. The authors validated the proposed model on both ultrasound and histopathology images. The experimental results demonstrated its promising performance. The other group focused on the prediction of radiation pneumonitis in patients with lung cancer based on lung perfusion functional images (Li et al.). The study first divided the whole lung volume into function-wise lung regions. Next, the authors extracted radiomics and dose features from function-wise and full lung regions, respectively. Finally, the authors proposed a multi-feature fusion model to fuse radiomics features, dose features, and combined dual-omics features. The experimental results demonstrated the promise of the function-wise dual-omics analysis method to improve the prediction of radiation pneumonitis in patients with lung cancer.

Additionally, three research groups designed a multimodal fusion algorithm, a registration system, and an identification system for personalized medicine in the field of brain diseases, and dental implants. Neuroimaging has been widely used as a precision diagnostic technique for brain diseases. Ran et al. collected 103 subjects with magnetic resonance imaging (MRI) and positron emission tomography (PET) data from the Alzheimer’s Disease Neuroimaging Initiative, and then proposed a multi-kernel model to fuse MRI and CT modalities for the diagnosis of Alzheimer’s disease. In the multi-kernel model, unlike the modality-consistent regularization used in previous studies, the authors designed a novel “all-single” fusion strategy that considers every single feature and the possible combinations, which allowed full exploration of the complementary information. Moreover, Ran et al. extended the compactness graph mechanism from the linear space to the multi-kernel space to reduce the overfitting problems in multi-kernel space. Image registration plays a significant role in the computational intelligence-assisted diagnosis of brain diseases. Kujur et al. proposed a general registration framework using the tissue P system for image registration. The innovation is that the proposed registration framework optimizes upon the mutual information similarity metric to identify a global solution. Kujur et al. evaluated the registration framework on single- and multi-modality brain image data collected from the Montreal Neurological Institute. The experimental results indicated that among all the state-of-the-art models, the proposed registration framework provided better mutual information values with minimum deviation in a range of experiment setups conducted iteratively. In the field of dental implants, identifying the appropriate accessories for installing a dental implant is a vital factor that impacts dental prosthesis sustainability and reliability. X-ray images are usually used to assist dentists in identifying the implant manufacturer to determine further treatment procedures. Guo et al. developed a dental implant identification system based on a novel thinner VGG (Visual Geometry Group) model with an on-demand client-server structure. The experimental results demonstrated the advantages of the proposed systems compared to state-of-the-art systems.

Another takeaway from this topic are two review manuscripts. Zhao et al. performed a systematic review on the application of computational intelligence in the diagnosis of ophthalmic disease. Specifically, the authors collected citation data from the Web of Science Core Collection database to evaluate the extent of the application of computational intelligence in the diagnosis of ophthalmic disease in publications between 1 January 2012 and 31 December 2021. They found that the hotspots of computational intelligence research on this topic have transitioned from the development of computational intelligence algorithms and the analysis of abnormal eye physiological structures to the investigation of more mature systems for ophthalmic disease diagnosis. The meta-analysis by Guo et al. analyzed the use of the transjugular intrahepatic portosystemic shunt (TIPS) for the prevention of rebleeding in patients with cirrhosis and portal vein thrombosis. The authors reported that TIPS is feasible and effectively prevents rebleeding in these patients, regardless of cavernous transformation of the portal vein.

In conclusion, this Research Topic mainly focused on computational intelligence in personalized medicine. Some representative works made use of classic computational intelligence technologies directly, while other representative works were based on newly proposed computational intelligence algorithms. In general, these works are excellent and open a new window for the development of computational intelligent algorithms in personalized medicine. Finally, we sincerely thank all the authors who contributed their work and provided articles, allowing us to coordinate and edit this outstanding collection.
